# Risk Factors for Esophageal Squamous Cell Carcinoma in Patients with Head and Neck Squamous Cell Carcinoma

**DOI:** 10.1155/2022/5227771

**Published:** 2022-08-27

**Authors:** Lei Wang, Wenjing Pang, Kun Zhou, Lei Li, Feng Wang, Wei Cao, Xiangjun Meng

**Affiliations:** ^1^Department of Gastroenterology, Shanghai Ninth Peoples' Hospital, Shanghai Jiao Tong University School of Medicine, Shanghai 200011, China; ^2^Center for Digestive Diseases Research and Clinical Translation of Shanghai Jiao Tong University, Shanghai 200011, China; ^3^Shanghai Key Laboratory of Gut Microecology and Associated Major Diseases Research, Shanghai 200011, China; ^4^Department of Thoracic Surgery, Shanghai Ninth Peoples' Hospital, Shanghai Jiao Tong University School of Medicine, Shanghai 200011, China; ^5^Department of Oral and Maxillofacial-Head and Neck Oncology, Shanghai Ninth Peoples' Hospital, Shanghai Jiao Tong University School of Medicine, Shanghai 200011, China; ^6^National Clinical Research Center for Oral Diseases, Shanghai 200011, China; ^7^Shanghai Key Laboratory of Stomatology & Shanghai Research Institute of Stomatology, Shanghai 200011, China; ^8^Research Unit of Oral and Maxillofacial Regenerative Medicine, Chinese Academy of Medical Sciences, Shanghai 200011, China

## Abstract

**Background:**

Esophageal squamous cell carcinoma (ESCC) is a common second primary neoplasia in patients with a history of head and neck squamous cell carcinoma (HNSCC). The aim of this study was to provide further information and novel insights into the risk factors for ESCC in patients with HNSCC.

**Methods:**

We retrospectively analyzed 98 HNSCC patients diagnosed from 2007 to 2017, 30 HNSCC patients suffering from ESCC, who had undergone endoscopic examination because of positive imaging examinations or symptoms, and 68 HNSCC patients who had no ESCC occurrence for at least six years post-HNSCC diagnosis. Associated clinicopathological data and lifestyle information of the ESCC group and the without ESCC group were collected, and a case-control study of risk factors was analyzed between the two groups.

**Results:**

The majority (83.4%) of the cases with HNSCC esophageal cancers were male patients over 50 years. We established that 93.75% (30/32) of the ESCC occurred within six years after HNSCC diagnosis. HNSCC location, stage, and radiotherapy history had no significant association with the development of ESCC. High Ki67 labeling index (Ki67 LI) (>46) patients tended to be 3.1 times (95% CI = 1.3–7.6) more likely to develop ESCC compared to low Ki67 LI (≤45) patients (*P* < 0.05). Drinkers with alcohol flushing response were at a 3.3 times higher risk to have ESCC (95% CI = 1.0–10.4) than drinkers without flush response (*P* < 0.05).

**Conclusions:**

HNSCC patients, especially drinkers with an alcohol flushing response, as well as those with high Ki67 LI of HNSCC tissue, were more likely to develop ESCC.

## 1. Introduction

Head and neck squamous cell carcinoma (HNSCC) patients have a high risk of developing second primary carcinoma in their upper gastrointestinal tract, most commonly in the esophagus [1, 2]. Reportedly, approximately 5%–15% of HNSCC patients develop esophageal squamous cell carcinoma (ESCC), which is higher than the general population [3–7]. Due to the usual absence of early clinical symptoms associated with esophageal malignant tumors, ESCC often leads to treatment failure in HNSCC, which is associated with a poor prognosis [8–10].

With advances in endoscopy, such as NBI in combination with magnifying endoscopy, early detection of esophageal cancer is possible. Thus, routine esophageal screening in asymptomatic patients with HNSCC has been recommended [11–17]. However, there is no consensus reached on the criteria for screening patients with a higher risk for esophageal cancer [18, 19]. Hence, a retrospective comparative case-control study between the ESCC group and the without ESCC group of HNSCC patients was conducted to investigate the associated risk factors of second primary ESCC in patients with HNSCC.

## 2. Materials and Methods

### 2.1. Study Design

We retrospectively reviewed the medical records of 98 HNSCC patients diagnosed for the first time at the Department of Oral and Maxillofacial Head and Neck Oncology, Shanghai Ninth Peoples' Hospital, Shanghai Jiaotong University (Shanghai, China), from 2007 to 2017. Thirty HNSCC patients suffering from synchronous or metachronous ESCC within or after six months following HNSCC diagnosis were included. Synchronous cancers were defined as the occurrence of a second primitive cancer within the first six months following the detection of first cancer, whereas metachronous cancers appeared within more than six months. Sixty-eight HNSCC patients without ESCC occurrence for at least six years after HNSCC diagnosis were included as a control group. The inclusion criteria were as follows: HNSCC patients were first diagnosed based on pathological postoperative confirmation, esophageal lesions were observed by upper gastrointestinal endoscopy with the NBI mode, and the histopathology of the esophageal lesions was confirmed as ESCC or high-grade intraepithelial neoplasia. The following exclusion criteria were applied: patients who had a history of esophageal cancer or other malignancies before HNSCC diagnosis, HNSCC patients who were not newly diagnosed in our hospital, and patients who had prior gastrointestinal surgery before HNSCC diagnosis. The study was conducted in accordance with the Declaration of Helsinki (as revised in 2013) and was approved by the Ethics Committee of Shanghai Ninth People's Hospital, Shanghai Jiao Tong University School of Medicine (No.: SH9H-2020-T189-1). Individual consent for this retrospective analysis was waived.

### 2.2. Information of HNSCC Patients

Associated clinicopathological data and lifestyle information of all HNSCC patients were collected. All HNSCC patients underwent surgical treatment at the Department of Oral and Maxillofacial Head and Neck Oncology, Shanghai Ninth Peoples' Hospital, Shanghai Jiao Tong University (Shanghai, China). Histopathological diagnoses were made at the department of oral pathology of our hospital. HNSCC was graded based on the surgical histopathological results and the TNM staging system for lip and oral cancer (AJCC, eighth edition) [20]. The Ki67 labeling index (Ki67 LI) was assessed based on immunohistochemical evaluation from histopathological reports of HNSCC operation tissues. IHC staining was performed following the standard procedure. Ki67-positive neoplastic epithelial cell counts were carried out in five randomly selected fields at 400 × magnification. Ki67 LI was derived as the number of Ki67-positive cells multiplied by 100 and divided by the total number of the observed neoplastic epithelial cells. The Ki67 antigen expression levels were categorized into the following categories: low, Ki67 LI ≤ 45; high, Ki67 LI > 45. Drinkers were defined as those consuming any alcoholic beverage at least once per week for a minimum of six months and smokers as those smoking ten cigarettes or more per week for at least six months [21]. Information on whether the drinkers experienced flushing in response to alcohol was collected. The radiotherapy history of each HNSCC patient was also reviewed.

### 2.3. Endoscopic Examination of Esophageal Cancer

Endoscopic examination was performed at the initial HNSCC diagnosis or at the postoperative follow-up based on positive imaging examination or the presence of symptoms such as progressive dysphagia or pain behind the sternum. Endoscopy of the upper gastrointestinal tract (GIF-H290; Olympus, Japan) was performed through the mouth by a physician experienced in endoscopy. Once in the oral cavity, the NBI mode was turned on for observation of the pyriform sinus and esophageal mucosa, focusing on any suspected esophageal lesions. Biopsy was next performed when a definitive pathological diagnosis was needed. Esophageal cancer was classified according to AJCC eighth edition guidelines [20].

### 2.4. Patient Follow-Up

All HNSCC patients were followed up by trained medical personnel by telephone or WeChat for follow-up treatment and a second primary cancer diagnosis. HNSCC patients were followed up to the time of esophageal cancer diagnosis or at least six years after HNSCC diagnosis.

### 2.5. Statistical Analysis

Comparisons between the ESCC group and the without ESCC group of HNSCC patients were performed using the *χ*^2^ test or Fisher's exact test. Logistic regression analysis was conducted using the data for sex, age, HNSCC site, HNSCC stage, Ki67 labeling, radiotherapy history, smoking history, drinking history, and flush response. Odds ratios and 95% confidence intervals were calculated. Statistical analysis was performed using GraphPad Prism 6. *P* values <0.05 were considered to indicate statistically significant differences.

## 3. Results

### 3.1. Characteristics of ESCC Cases in HNSCC Patients

Thirty HNSCC patients suffering from ESCC were included in this study. Of them, two HNSCC patients had recurrent cases of ESCC. The average interval time between ESCC and HNSCC diagnosis was 36.0 ± 39.2 months; the longest interval time was 180 months. There were 8 (25%) cases of synchronous carcinoma with an interval time within 6 months, 13 (40.6%) cases from 6 months to 3 years, 9 (28.1%) cases from 3 years to 6 years, and 2 (6.3%) case more than 6 years. Overall, 93.75% (30/32) of ESCC occurred within 6 years after HNSCC diagnosis.

Тhe most common ESCC location was in the middle thoracic segment of the esophagus (13 cases) (40.6%), followed by 9 (28.1%) cases in the upper thoracic esophagus, 7 (21.9%) in the lower thoracic esophagus, and 3 (9.4%) in the cervical esophagus. There were 5 (15.6%) cases with early ESCC and 27 (84.4%) cases with advanced ESCC. In terms of endoscopic morphology, 1 (3.1%) case of early ESCC was 0-Is type and 4 (12.5%) cases were 0-IIb type, and the 27 cases of advanced ESCC were mass type (7 cases) (21.9%), mass infiltration type (10 cases) (31.3%), ulcer type (1 case) (3.1%), ulcer infiltration type (6 cases) (18.8%), and constriction type (3 cases) (9.4%). The endoscopic morphology of ESCC consisted mainly of the mass and mass infiltration types (Table 1).

### 3.2. Risk Factors Associated with ESCC Occurrence in HNSCC Patients

The case-control study was performed on HNSCC patients suffering from synchronous or metachronous ESCC and HNSCC patients without ESCC occurrence. Ninety-eight HNSCC patients were retrospectively reviewed in this research, 30 HNSCC patients with ESCC were enrolled in the ESCC group, and 68 HNSCC patients were in the without ESCC group. Although no significant differences in gender and age were observed between the ESCC and the without ESCC groups, of the 30 HNSCC patients with ESCC, 25 (83.4%) were over 50 years, and 28 (93.3%) of the patients were male. We found that male patients were 3.3 times (95% CI = 0.7–15.4) more likely to develop ESCC than female patients. The risk remained to be 1.3 times (95% CI = 0.4–3.7) and 2.1 times (95% CI = 0.6–6.8), respectively, as the age of patients increased by 10 years.

**Figure 1:**
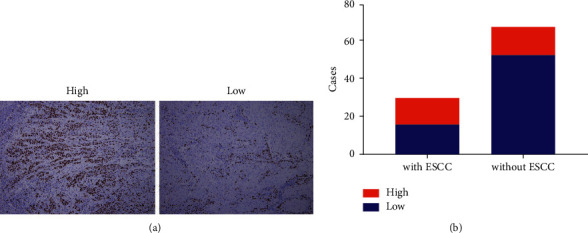
Ki67 immunohistochemical expression (Ki67 labeling index, Ki67 LI) in HNSCC operation tissues with or without secondary ESCC. (a) High Ki67 LI (>45) and low Ki67 LI (≤45) in HNSCC operation tissues (immunohistochemistry; magnification ×100). (b) Bar plots representing high Ki67 LI in HNSCC with ESCC patients compared to HNSCC without ESCC patients.

Concerning the clinicopathological risk factors, the most common HNSCC location in the ESCC group was the tongue (33.3%), followed by the soft palate (23.3%), the mouth floor (20.0%), gingiva (16.7%), and the lip and the larynx (3.3%), with no significant differences in the HNSCC location distribution of the without ESCC group. About the HNSCC stage, for the ESCC group, there were 10 (33.3%) cases with stage I, 7 (23.3%) cases with stage II, 8 (26.7%) cases with stage III, and 5 (16.7%) cases with stage IV. In the without ESCC group, there were 26 (38.2%) cases with stage I, 15 (22.1%) cases with stage II, 14 (20.6%) cases with stage III, and 13 (19.1%) cases with stage IV of the disease. The HNSCC stage was not significantly associated with ESCC development. Ki67 immunohistochemical expression was analyzed in specimens of HNSCC operation tissues. The results showed that in the ESCC group, 16 (53.3%) cases had low Ki67 LI (≤45) and 14 (46.7%) cases had high Ki67 LI (>46). In the without ESCC group, 53 (77.9%) cases had low Ki67 LI and 15 (22.1%) cases had high Ki67 LI. HNSCC patients with ESCC tended to have significantly higher Ki67 LI levels than HNSCC patients without ESCC (*P* < 0.05). Additionally, high Ki67 LI (>46) patients tended to be 3.1 times (95% CI = 1.3–7.6) more likely to develop ESCC than low Ki67 LI (≤45) patients (Figure 1). We established that 14 (46.7%) HNSCC with ESCC patients and 40 (58.8%) HNSCC without ESCC patients underwent postoperative radiotherapy, with no significant differences in radiotherapy history between the two groups.

Regarding lifestyle risk factors, smoking was not significantly associated with the occurrence of ESCC. Alcohol drinkers were 1.9 times (95% CI = 0.7–4.8) more likely to develop ESCC than nondrinkers, despite the lack of a statistically significant difference. Furthermore, drinkers with alcohol flushing response were more likely to develop ESCC than drinkers without flush response (*P* < 0.05). HNSCC patients were exposed to a 3.3 times higher risk to develop ESCC (95% CI = 1.0–10.4) if they had alcohol flush response (Table 2).

## 4. Discussion

In recent years, field cancerization has attracted considerable research attention [22–24]. Esophageal and oral mucosa epithelial cells are both squamous epithelial cells, which are exposed to similar environments. Hence, HNSCC patients are at high risk for second primary carcinoma of ESCC [25]. Previous studies established a 5%–15% incidence of esophageal cancer in HNSCC patients, which was significantly higher than that in the general population [3–7].

We found that the period of ESCC manifestation was large, ranging from concurrence with HNSCC to many years after HNSCC. However, 93.75% (30/32) of the ESCC cases occurred within six years after HNSCC diagnosis. Due to the long timespan of second primary ESCC, the selection of HNSCC patients and targeted upper gastrointestinal endoscopy are critical to the improvement of the early diagnosis rate of synchronous and metachronous ESCC. To clarify the associated risk factors of ESCC in patients with HNSCC, we retrospectively analyzed HNSCC cases with ESCC who were diagnosed by upper gastrointestinal endoscopy in our hospital from 2007 to 2017. A case-control study was performed with these patients without ESCC occurrence for at least six years after HNSCC diagnosis.

Of the 30 HNSCC patients with recurrent ESCC in our present study that developed esophageal secondary primary cancer, 25 (83.4%) were aged over 50 years and 28 (93.3%) were male. Despite the lack of significant differences between the two groups included in our study, male and old-age HNSCC patients were at a higher risk to develop ESCC. These results may be due to the limited sample size of our investigation and the fact that HNSCC and ESCC patients had similar characteristics in terms of gender and age. Previous reports showed that older age (over 65 years) was significantly higher in patients with second primary esophageal cancer than in patients without esophageal cancer [26].

Our case-control study revealed that HNSCC cases with ESCC and without ESCC were similar and most commonly in the tongue and soft palate. Other research findings established no association between esophageal cancers and HNSCC location [27]. However, a larger number of esophageal secondary primary cancers were reportedly detected in patients with hypopharyngeal and oropharyngeal cancers [1]. The reasons for the different outcomes in these localizations remain to be elucidated, partly because the conclusions might have been obscured by the small sample size and the approaches employed to locate HNSCC. Furthermore, we found no significant relationship between ESCC recurrence and HNSCC staging. However, an earlier study showed that patients with esophageal recurrent cancer had earlier HNSCC staging, which may be because HNSCC patients with earlier stages had adequate time to develop recurrence [28].

Ki67 is a large nonhistone protein present in the nucleus and nucleolar region, which is observed in proliferating cells. The expression of Ki67 is a diagnostic and poor prognostic biomarker for HNSCC patients [29]. Here, we established that the Ki67 LI was significantly higher in HNSCC patients with ESCC. Therefore, it may be suggested that quantitative measurements of Ki67-positive neoplastic epithelial cells in HNSCC can be used to reveal the potential risks of recurrent ESCC. Further research is recommended with a large cohort of HNSCC patients to validate the predictive value of Ki67 LI.

The consumption of alcohol and cigarettes is a significant risk factor for HNSCC and esophageal cancer [30, 31]. A prospective study found that high-dose drinkers of HNSCC with a flush reaction were at a significantly higher risk of developing synchronous ESCC; drinkers with a higher daily average or cumulative amount were also exposed to a significantly higher risk for the development of ESCC [4]. Similar to previous studies, the present study further confirmed that alcohol flushing response is a risk factor of ESCC in patients with HNSCC. We also found that the risk for ESCC development in alcohol drinkers was 1.9 times higher than that in nondrinkers, but the impact of the cumulative dose of alcohol consumption was difficult to clarify in this retrospective study. Alcohol flushing response is associated with inherited deficiencies in the enzyme aldehyde dehydrogenase 2 (ALDH2), resulting in accumulation of acetyl aldehyde. The East Asian-specific dysfunctional ALDH2*∗*2 missense mutation is a genetic risk factor for upper aerodigestive tract (UADT) cancer. An earlier epidemiological study clearly showed that ALDH2*∗*2 was associated with increased susceptibility to synchronous and metachronous UADT cancers and was highly represented in the majority of such cancer patients, who also had faster cancer progression and poor prognosis [32].

## 5. Conclusions

In the present study, we found that HNSCC patients, especially drinkers with an alcohol flushing response, as well as those with high Ki67 LI in their HNSCC tissues were more likely to develop ESCC. Nevertheless, the conclusions that can be drawn have limited representativeness to make as this study was retrospective and with a very small sample size. Therefore, further prospective research is needed to confirm our present findings.

## Figures and Tables

**Table 1:** Characteristics of ESCC cases in HNSCC patients.

	ESCC cases (*n* = 32), *N* (%)
Interval time (months)	
≤6 months	8 (25)
6 months–3 years	13 (40.6)
3–6 years	9 (28.1)
>6 years	2 (6.3)

ESCC location	
Neck	3 (9.4)
Upper thoracic	9 (28.1)
Middle thoracic	13 (40.6)
Lower thoracic	7 (21.9)

ESCC stage	
Early	5 (15.6)
Developed	27 (84.4)

Endoscopic morphology	
Early	
0-Is	1 (3.1)
0-IIb	4 (12.5)
Developed	
Mass type	7 (21.9)
Mass infiltration type	10 (31.3)
Ulcer type	1 (3.1)
Ulcer infiltration type	6 (18.8)
Peripheral constriction type	3 (9.4)

**Table 2:** Risk factors associated with ESCC occurrence in HNSCC patients.

	With ESCC (*n* = 30), *N* (%)	Without ESCC (*n* = 68), *N* (%)	Total (*n* = 98), *N* (%)	*P*	OR (95% CI)
Sex				0.1390	
Female	2 (6.7)	13 (19.1)	15 (15.3)		1
Male	28 (93.3)	55 (80.9)	83 (84.7)		3.3 (0.7, 15.4)

Age (years)				0.4618	
40–50	5 (16.7)	16 (23.5)	21 (21.4)		1
51–60	14 (46.7)	35 (51.5)	49 (50.0)		1.3 (0.4, 3.7)
>60	11 (36.7)	17 (25.0)	28 (28.6)		2.1 (0.6, 6.8)

HNSCC site				0.7129	
Lip	1 (3.3)	5 (7.4)	6 (6.1)		1
Gingiva	5 (16.7)	8 (11.8)	13 (13.3)		3.1 (0.3, 43.3)
Larynx	1 (3.3)	7 (10.3)	8 (8.2)		0.7 (0.0, 16.1)
Mouth floor	6 (20.0)	12 (17.6)	18 (18.4)		2.5 (0.3, 33.8)
Soft palate	7 (23.3)	19 (27.9)	26 (26.5)		1.8 (0.3, 24.5)
Tongue	10 (33.3)	17 (25.0)	27 (27.6)		2.9 (0.4, 37.7)

HNSCC stage				0.9051	
Stage I	10 (33.3)	26 (38.2)	36 (36.7)		1
Stage II	7 (23.3)	15 (22.1)	22 (22.4)		1.2 (0.4, 4.1)
Stage III	8 (26.7)	14 (20.6)	22 (22.4)		1.5 (0.5, 4.9)
Stage IV	5 (16.7)	13 (19.1)	18 (18.4)		1.0 (0.3, 3.2)

Ki67 labeling index				0.0177	
Low (≤45)	16 (53.3)	53 (77.9)	69 (70.4)		1
High (>45)	14 (46.7)	15 (22.1)	29 (29.6)		3.1 (1.3, 7.6)

Radiotherapy				0.2802	
No	16 (53.3)	28 (41.2)	44 (44.9)		1
Yes	14 (46.7)	40 (58.8)	54 (55.1)		0.6 (0.3, 1.5)

Smoking habits				0.7727	
Nonsmoker	5 (16.7)	13 (19.1)	18 (18.4)		1
Smoker	25 (83.3)	55 (80.9)	80 (81.6)		1.2 (0.4, 3.3)

Drinking habits				0.2452	
Nondrinker	7 (23.3)	25 (36.8)	32 (32.7)		1
Drinker	23 (76.7)	43 (63.2)	66 (67.3)		1.9 (0.7, 4.8)

Flush				0.0401	
Without flush	6 (20.0)	23 (33.8)	29 (29.6)		1
With flush	17 (56.7)	20 (29.4)	37 (37.8)		3.3 (1.0, 10.4)

## Data Availability

No data were used to support this study.

## Additional Points

The authors have completed the STROBE reporting checklist.
